# Supplementary material for: Amorphization-induced surface electronic states modulation of cobaltous oxide nanosheets for lithium-sulfur batteries
Source: Nat Commun. 2021 May 25;12:3102. doi: 10.1038/s41467-021-23349-9 (PMC8149689; doi:10.1038/s41467-021-23349-9)
Supplement: Supplementary file 1 — Supplementary Information [file 41467_2021_23349_MOESM1_ESM.pdf]

Supplementary Information

Amorphization-Induced Surface Electronic States Modulation of Cobaltous Oxide Nanosheets for Lithium-Sulfur Batteries

Li *et al.*

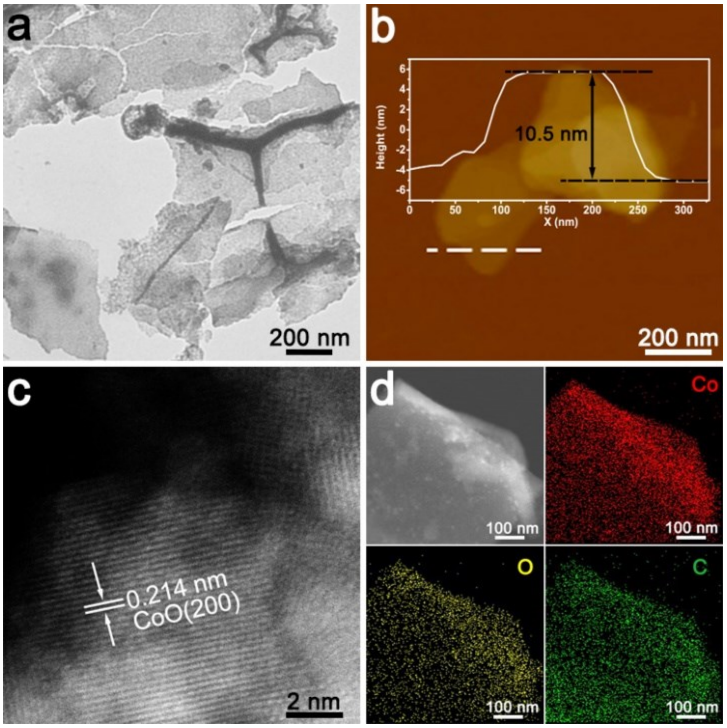

Supplementary Figure 1. (a) TEM image of c-CoO NSs. (b) AFM image of c-CoO NSs. (c) HAADF-STEM image of c-CoO NSs. Clear lattice fringes are observable in c-CoO NSs with distance of 0.214 nm corresponding to the (200) crystal facets of crystalline CoO. (d) HAADF-STEM image of c-CoO NSs and corresponding element maps showing the distribution of O (yellow), Co(red) and C (green).

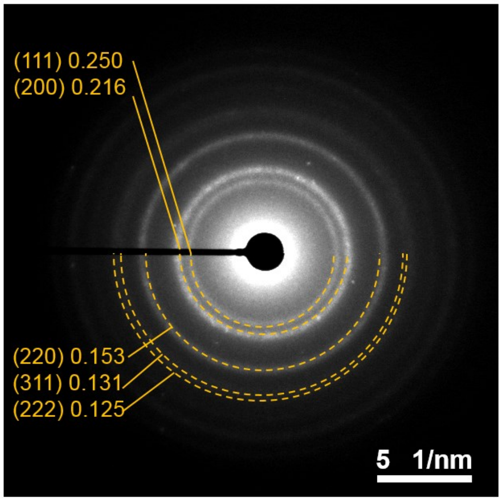

Supplementary Figure 2. Electron diffraction (ED) of c-CoO NSs. ED pattern of c-CoO NSs displays five distinct diffraction rings, which are indexed to the (111), (200), (220), (311) and (222) planes of the cubic Fm$\bar{3}$m phase of CoO poly-crystallites, respectively, and well consistent with the XRD results.

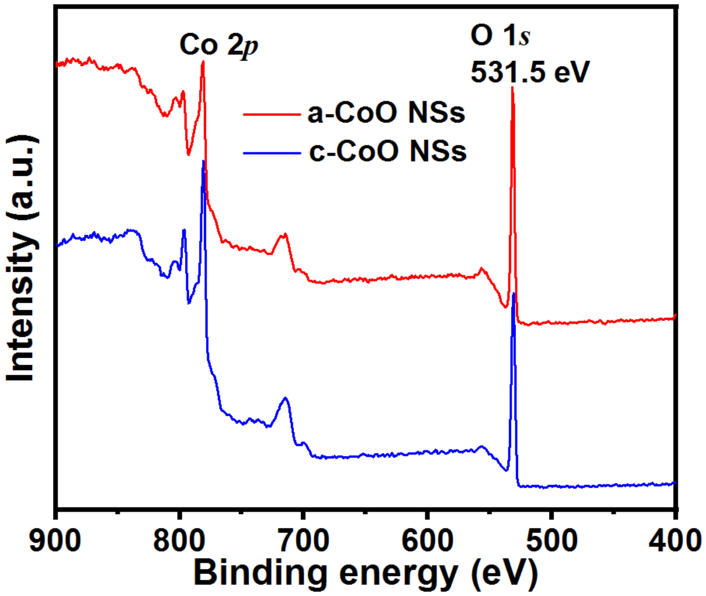

Supplementary Figure 3. XPS survey spectra of the a-CoO NSs and the c-CoO NSs.

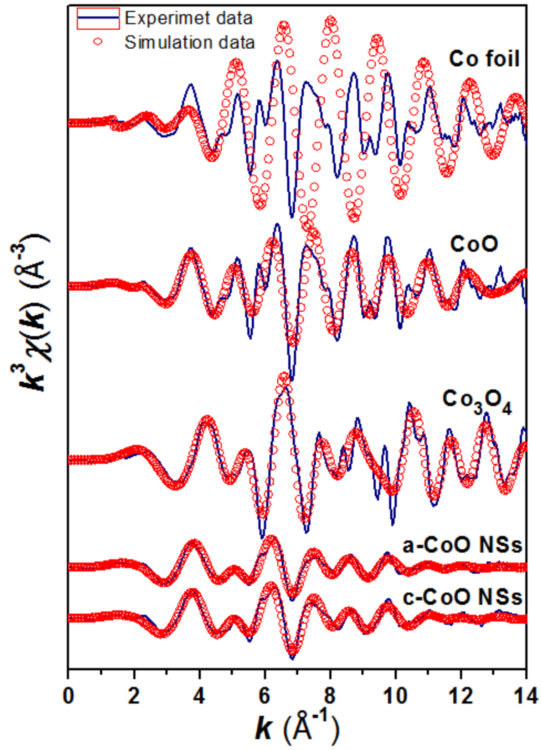

Supplementary Figure 4. The corresponding EXAFS fitting of a-CoO NSs, c-CoO NSs, Co_3O_4, CoO samples and Co foil at k space.

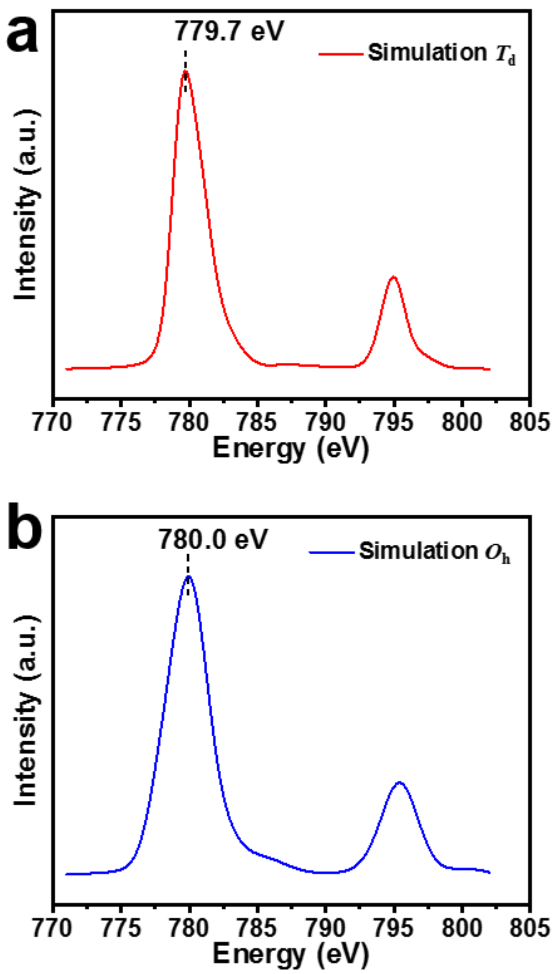

Supplementary Figure 5. Co $L_{2,3}$-edge XANES spectrum of the (a) simulation of Co^{2+} T_d sites and (b) simulation of Co^{2+} O_h sites. The adjusted parameters of the simulation by CTM4XAS are given in Supplementary Table 2.

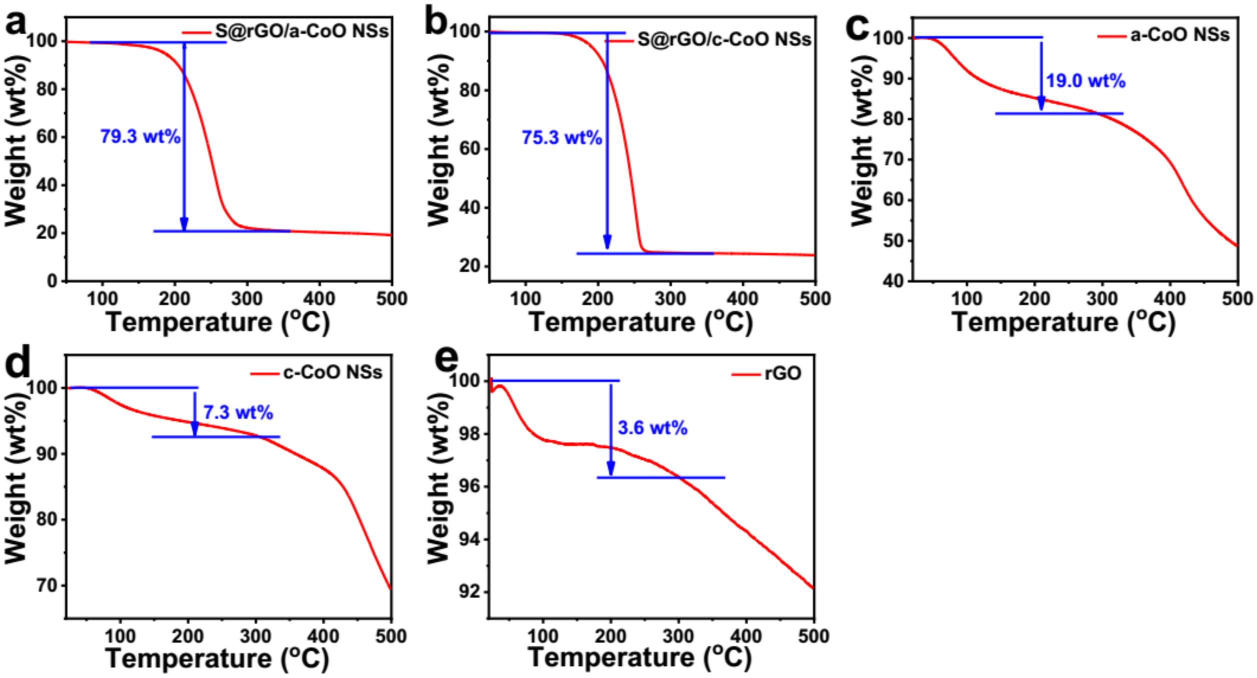

Supplementary Figure 6. Thermogravimetric (TG) analysis of the (a) S@rGO/a-CoO NSs, (b) S@rGO/c-CoO NSs, (c) a-CoO NSs, (d) c-CoO NSs and (e) rGO. Sulfur was loaded into rGO, rGO/a-CoO NSs and rGO/c-CoO NSs by a melt-diffusion method in a glass tube with controlled weight ratios (rGO : a-CoO NSs/c-CoO NSs = 4:1). The added sulfur had a weight ratio of 4:1 to the rGO/a-CoO NSs and the rGO/c-CoO NSs. Thus, the loading content of S is approximately equal to the weight loss rate in the TGA of the S@rGO/a-CoO NSs electrode minus 4% times the mass loss rate of the a-CoO NSs and 16% times the mass loss rate of the rGO at 300 °C. It was calculated that the loading content of S in the S@rGO/a-CoO NSs electrode was 78.0wt%. Similarly, the loading content of S in S@rGO/c-CoO NSs electrode is 74.4wt%.

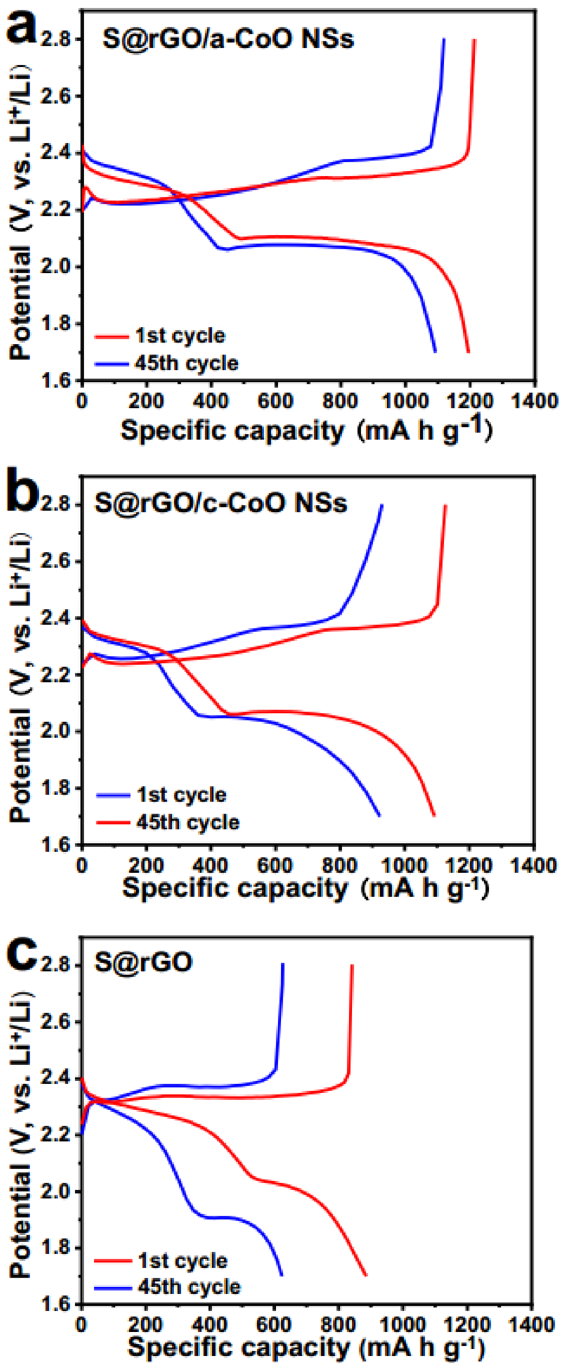

Supplementary Figure 7. Discharge-charge voltage curves before and after rate capability tests for (a) S@rGO/a-CoO NSs electrode at 0.5 C, (b) S@rGO/c-CoO NSs electrode at 0.5 C and (c) S@rGO electrode at 0.5 C.

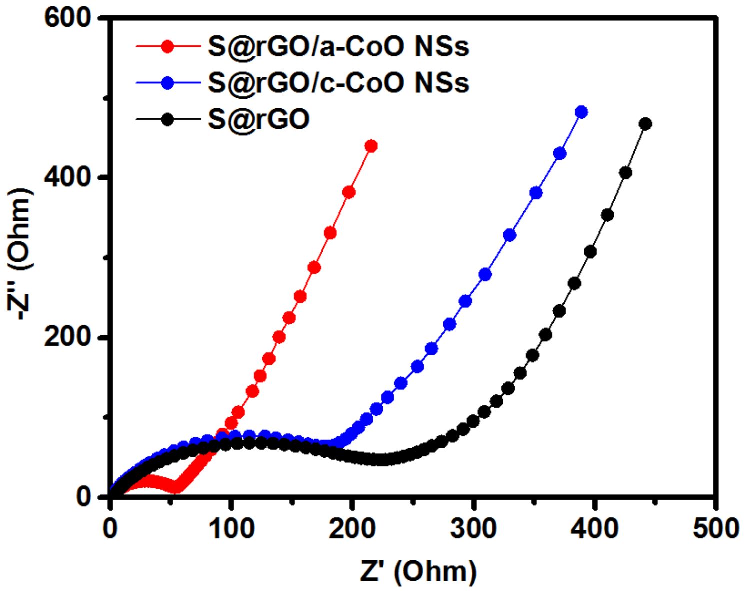

Supplementary Figure 8. Nyquist plots of S@rGO/a-CoO NSs, S@rGO/c-CoO NSs and S@rGO battery respectively.

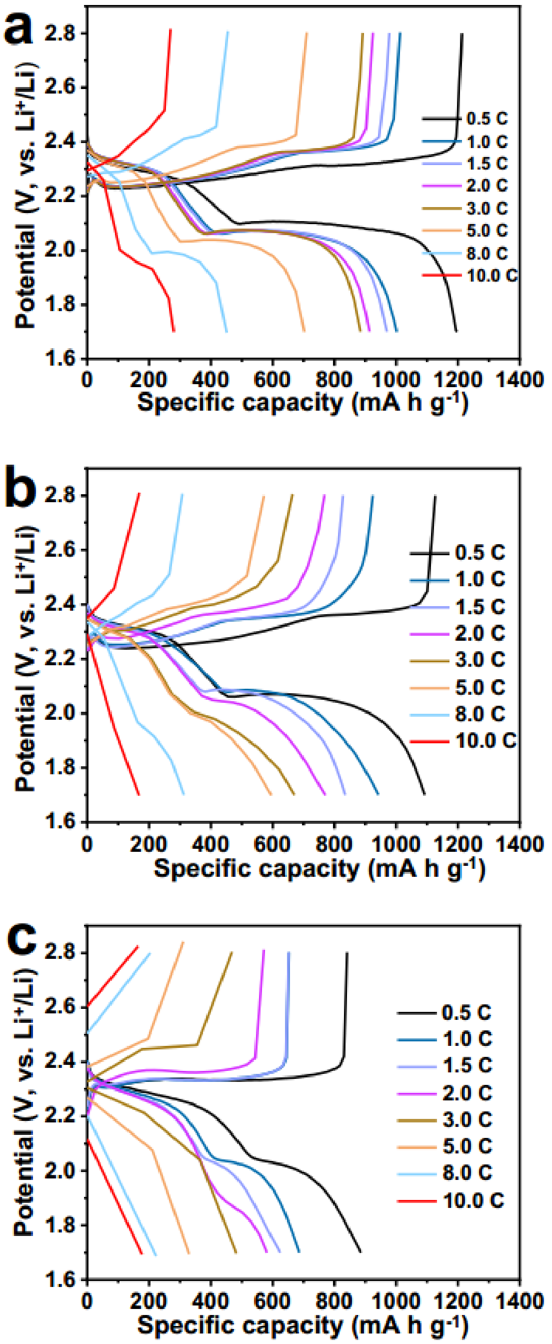

Supplementary Figure 9. Discharge-charge voltage curves of (a) S@rGO/a-CoO NSs, (b) S@rGO/c-CoO NSs and (c) S@rGO electrodes at various rates from 0.5 to 10.0 C.

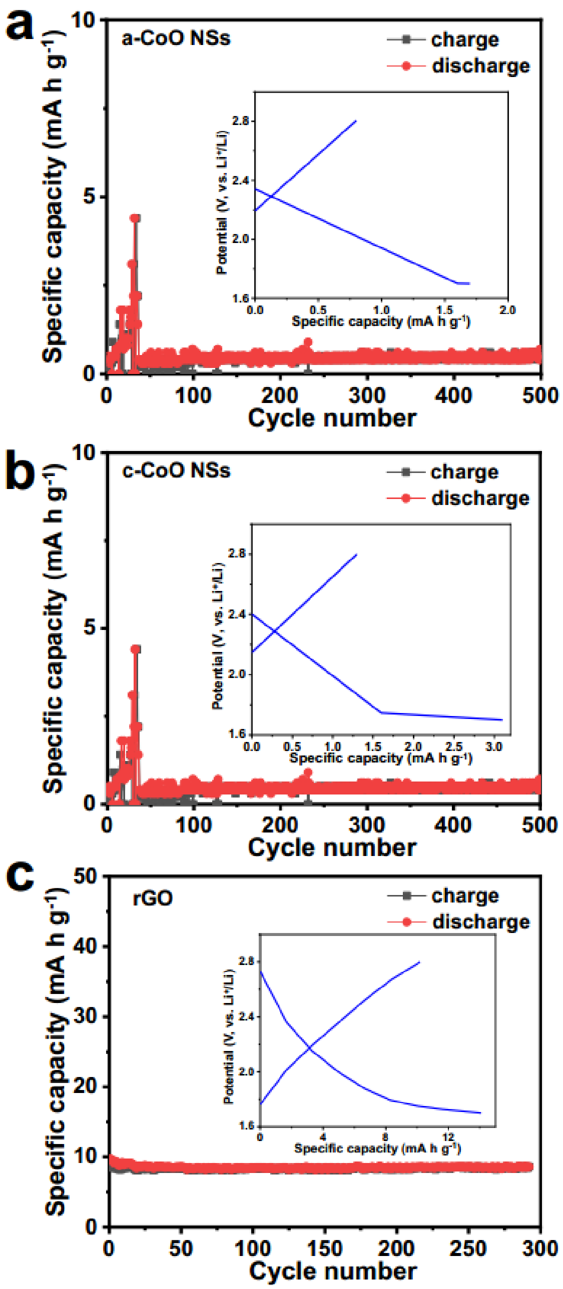

Supplementary Figure 10. The electrochemical performance of (a) a-CoO NSs, (b) c-CoO NSs and (c) rGO electrodes without sulfur, (insets: discharge-charge voltage curves of the three electrodes, respectively). Clearly, their capacity contributions are quite small, in comparison with the sulfur.

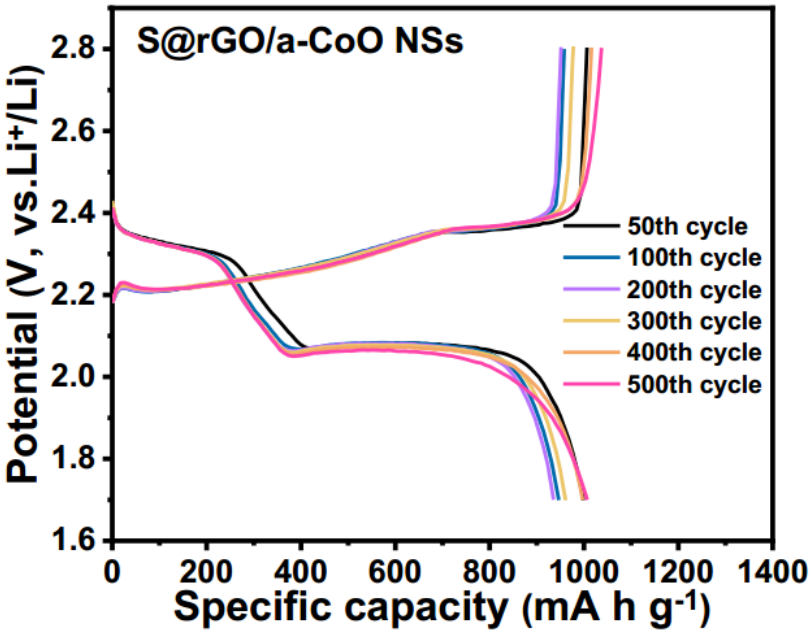

Supplementary Figure 11. Discharge-charge voltage curves of long-term cycles for S@rGO/a-CoO NSs electrode at 1 C.

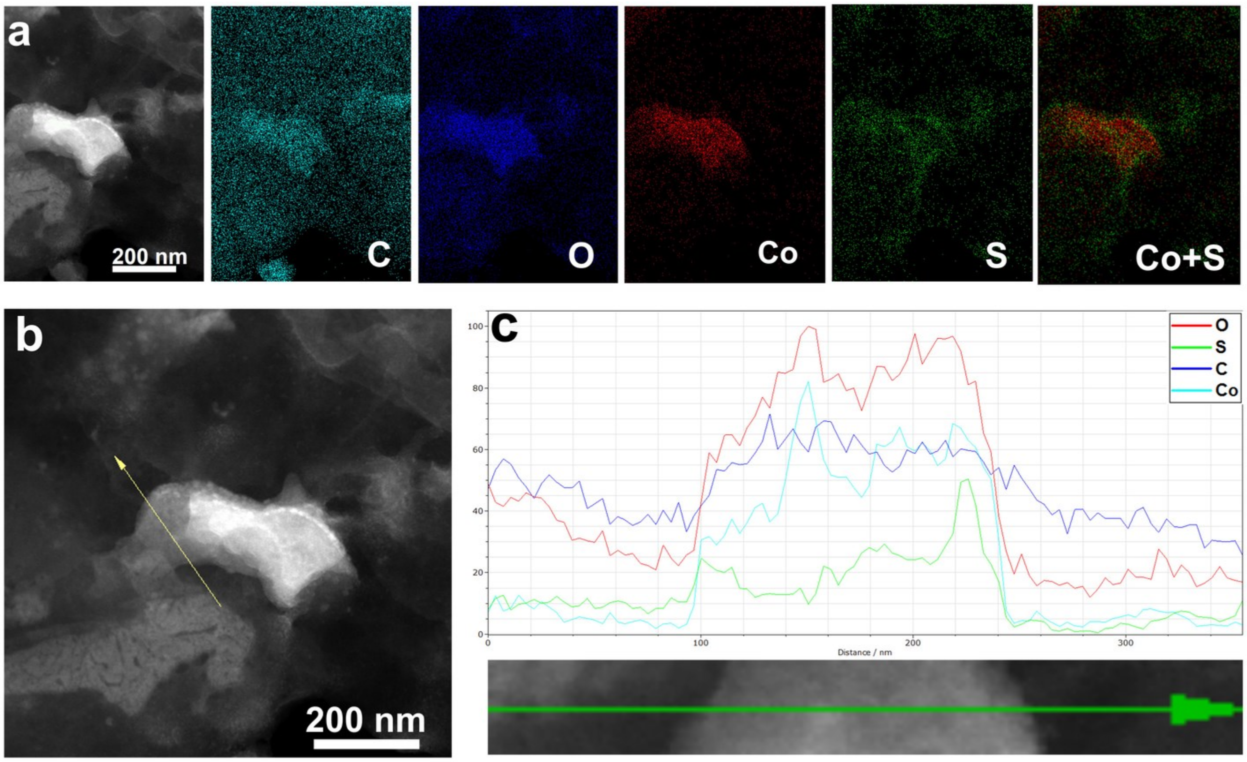

Supplementary Figure 12. (a) HAADF-STEM and the corresponding element mappings images, (b) and (c) EDS line scans of S@rGO/a-CoO NSs in the middle of discharge. The average atomic ratio of S:Co element in the CoO region is 0.49.

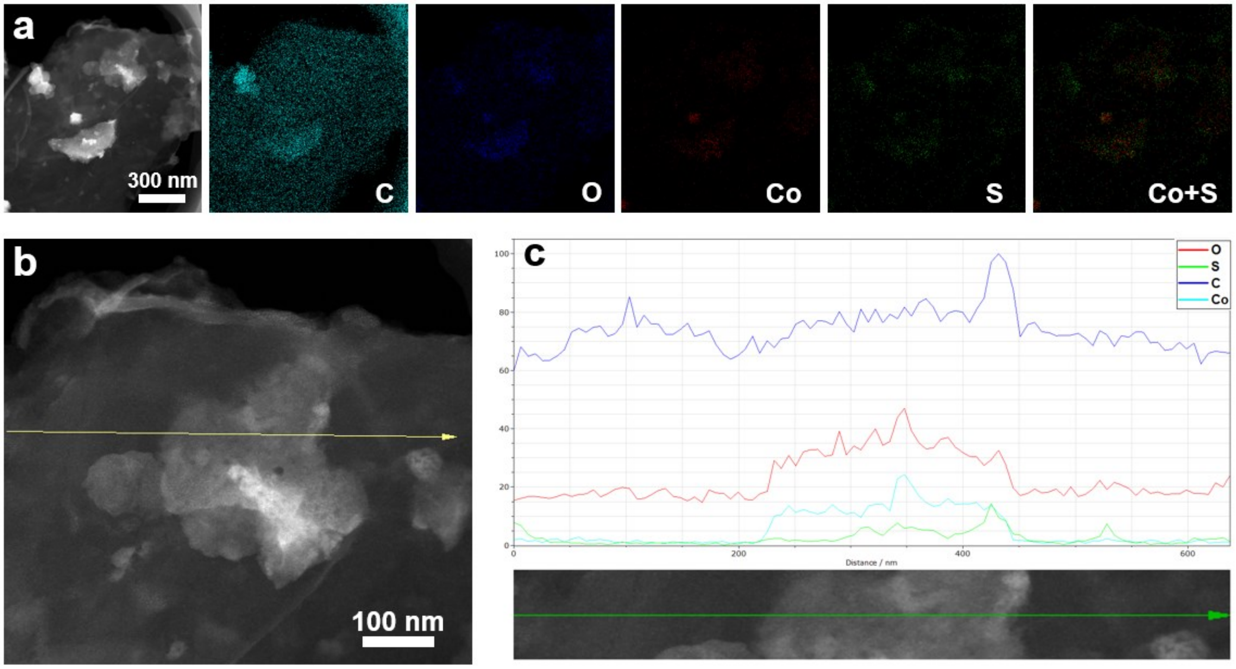

Supplementary Figure 13. (a) HAADF-STEM image and the corresponding element mappings images, (b) and (c) EDS line scans of S@rGO/c-CoO NSs in the middle of discharge. The average atomic ratio of S:Co element in the CoO region is 0.35.

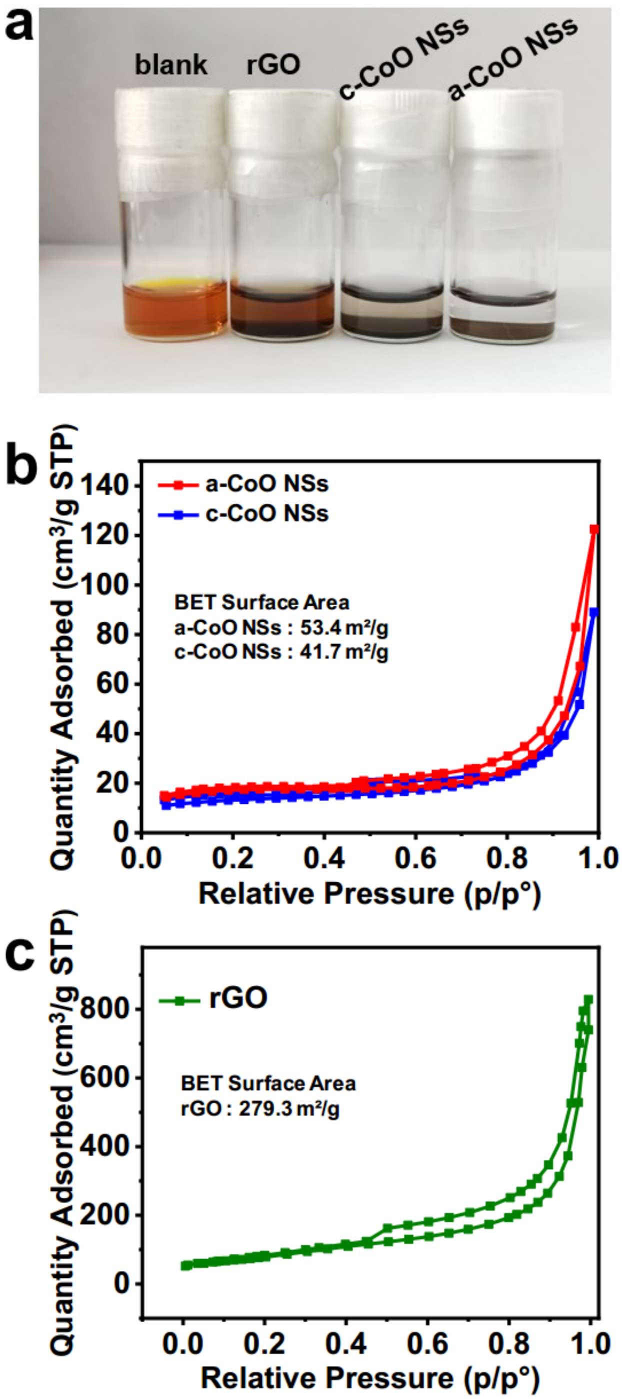

Supplementary Figure 14. (a) Visualized adsorption of Li_2S_6 by pristine rGO, a-CoO NSs and c-CoO NSs with the same total surface area. N_2 adsorption-desorption curves and analysis of (b) a-CoO NSs and c-CoO NSs and (c) rGO. BET surface area of a-CoO NSs, c-CoO NSs and rGO is 53.4 m^2/g, 41.7 m^2/g and 279.3 m^2/g respectively.

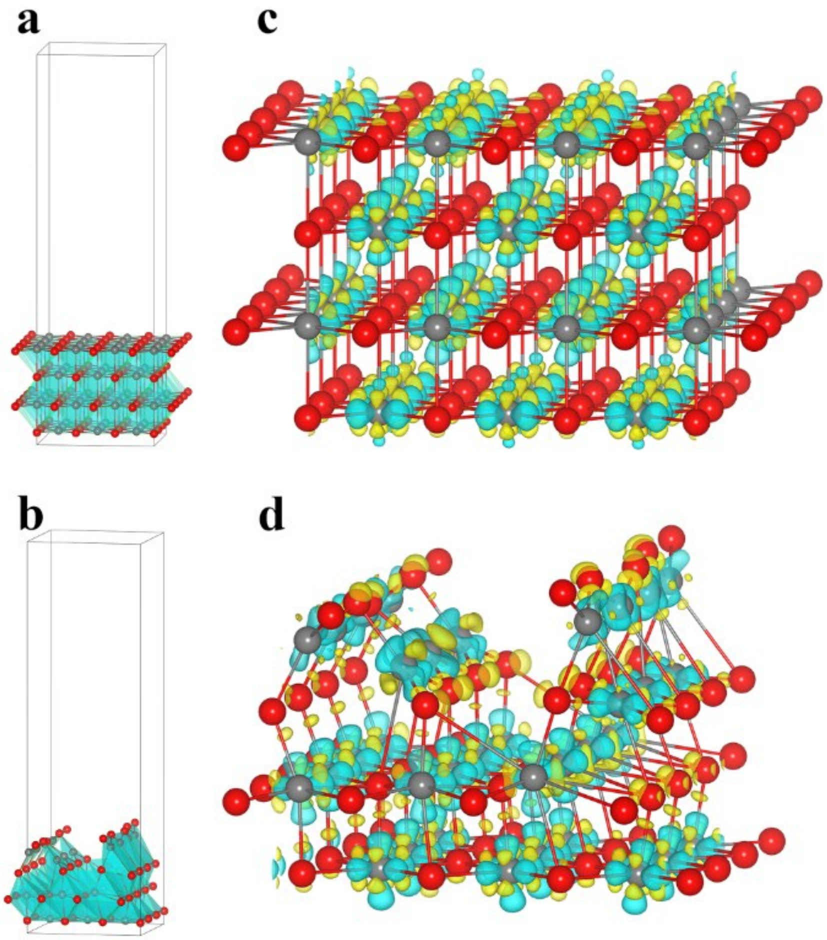

Supplementary Figure 15. The optimized structures of (a) c-CoO, (b) a-CoO; the deformation charge density of (c) c-CoO and (d) a-CoO. Red balls: O; grey balls, Co; yellow areas, charge accumulation; navy area, charge depletion.

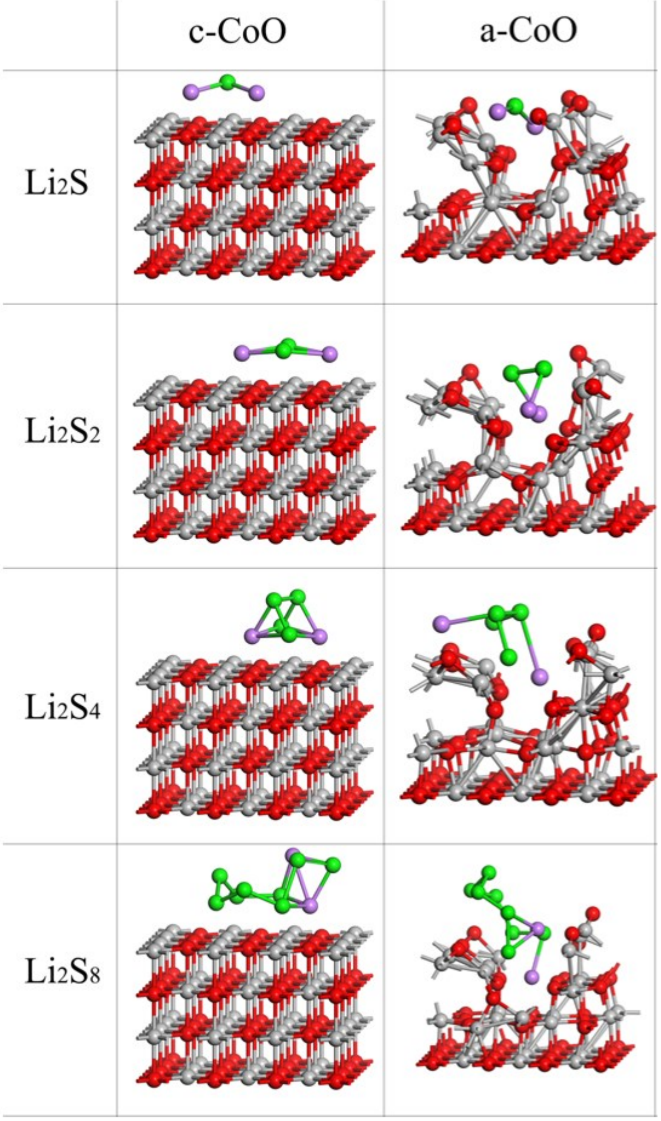

Supplementary Figure 16. The optimized structures of Li_2S_x on c-CoO and a-CoO. Red balls: O; grey balls, Co; purple balls: Li; green balls, S.

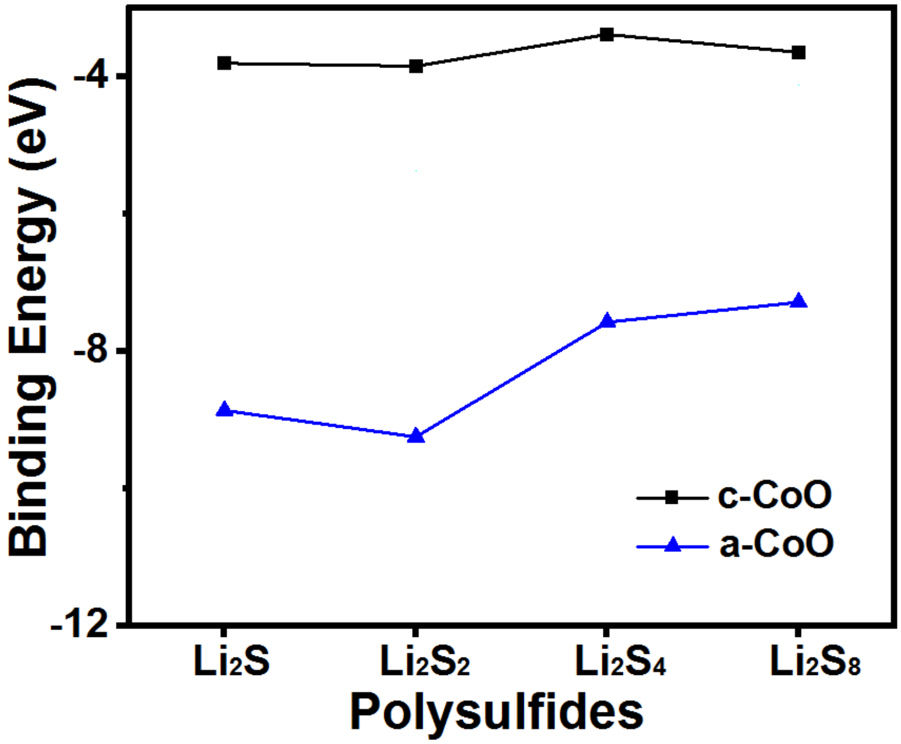

Supplementary Figure 17. The binding energy of Li_2S_x on c-CoO and a-CoO surfaces.

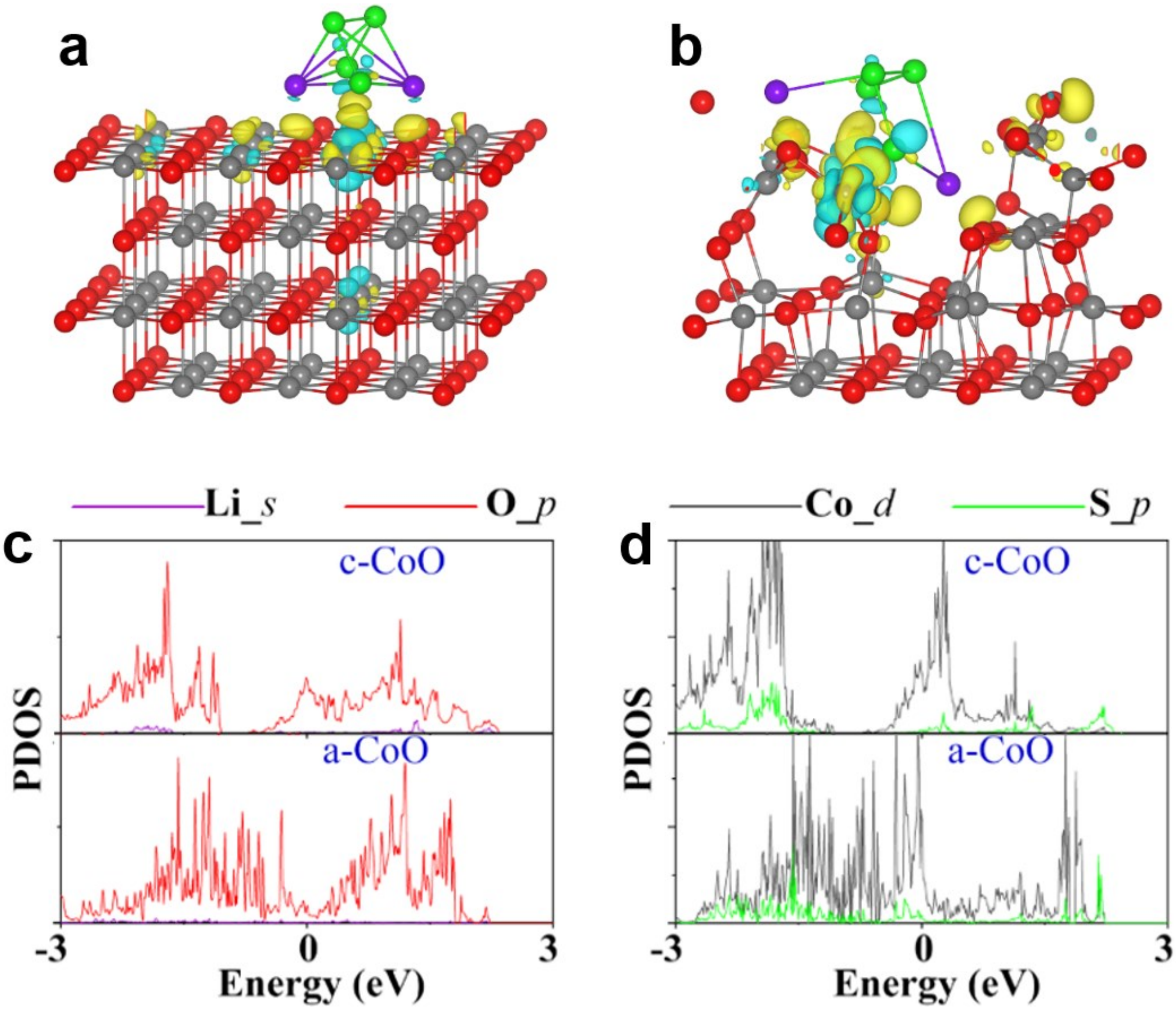

Supplementary Figure 18. Charge density difference for Li_2S_4 adsorbed (a) c-CoO and (b) a-CoO. Red balls: O; grey balls, Co; purple balls: Li; green balls: S; yellow areas: charge accumulation; navy area: charge depletion. Projected Density of State (PDOS) of (c) Li, O atoms and (d) Co, S atoms for Li_2S_4 on c-CoO and a-CoO.

Supplementary Table 1. EXAFS fitting parameters at the Co K-edge for various samples （S_0^2=0.776）

Sample	Shell	N^a	R(Å)^b	σ^2(Å^2)^c	ΔE_0 (eV)^d	R factor
Co foil	Co-Co	12	2.49	0.0063	7.2	0.0004
CoO	Co-O	6.0	2.07	0.0156	-3.2	0.0004
	Co-Co	13.3	3.00	0.0094		
Co_3O_4	Co-O	5.7	1.92	0.0035	-0.7	0.0004
	Co-Co	5.7	2.86	0.0055		
	Co-Co	7.0	3.37	0.0055		
a-CoO NSs	Co-O	5.5	2.05	0.0109	-2.9	0.0026
	Co-Co	8.2	3.02	0.0147		
c-CoO NSs	Co-O	5.8	2.03	0.0104	-5.5	0.0004
	Co-Co	10.1	2.99	0.0148		

$^a N$: coordination numbers; $^b R$: bond distance; $^c\sigma^2$: Debye-Waller factors; ^{d} ΔE_0: the inner potential correction. R factor: goodness of fit. S_0^2 was set to 0.776, according to the experimental EXAFS fit of Co foil reference by fixing CN as the known crystallographic value.

The XAFS data were analyzed using the software packages Demeter.[1] The spectra were normalized using Athena firstly, and then shell fittings were performed with Artemis. The χ (k) function was Fourier transformed (FT) using k^3 weighting, and all fittings were done in R-space. The amplitude reduction factor (S_0^2) was estimated to be 0.776 according to the fitting results of the cobalt foil. The coordination parameters of sorption samples were obtained by fitting the experimental peaks with theoretical amplitude.

Supplementary Table 2. The adjusted parameters for the simulation of Co L-edge XANES spectra by CTM4XAS (units: eV).

**Site symmetry**	**Crystal field strength (10Dq)**	**Charge transfer energy (Δ)**	**Hopping e_g electrons (t_e)**	**Hopping t_{2g} electrons (t_t)**	**Hubbard U value (U_{dd})**	**Core hole potential (U_{pd})**
Co^{2+} O_h	1.0	1.0	2.0	1.0	4.0	5.0
Co^{2+} T_d	-0.5	6.0	1.0	2.0	4.0	5.5

Details on the applied screening parameters: The Slater integrals including F_{dd}, F_{pd} and G_{pd} are all set to 1.0 as default. The core (2*p*) spin-orbit coupling and the valence (3*d*) spin-orbit coupling are both set to 1.0 as default.

Supplementary Table 3. Gaussian function value *c* for various simulations.

sample	symmetry	*c*
a-CoO NSs	O_h	0.95
	T_d	0.60
c-CoO NSs	O_h	0.95

Details on the applied convolution of transition intensities: Lorentzian broadening of 0.2 eV half-width half-maximum was applied in each calculated transition intensity to simulate the lifetime broadening of the core hole. And then the curve was convolved with a Gaussian function to simulate the energy resolution of the beamline and other mechanisms of spectral line broadening. The values of *c* in the Gaussian function were obtained by fitting, together with the ratio of O_h and T_d symmetries.

Supplementary Table 4. The electrochemical performance comparison with typical metal oxides or cobalt-based sulfur cathodes ever reported.

**Samples**	**Content of Sulfur** (wt%)	**Current density (C)**	**Initial capacity (mAh g^{-1})**	**Cycle number**	**Reversible capacity (mAh g^{-1})**	**reference**
S@rGO/a-CoO NSs	78.0	1	1248.2	500	1037.3	This work
CoO/ HCN–S	71.3	0.2	1242	200	996	2
CC@Co_3O_4	/	2	621	500	476	3
Co_3O_4-S	~60	0.2	1167	200	656	4
MnO_2@HCF/S	71	0.5	890	300	662	5
CNT/$NiFe_2O_4$-S	~76	1	~900	500	850	6
Co_9S_8/S75	75	2	~857	400	643	7
$CoSe_2$/G	63	4	984	500	503	8
S@Fe_3O_4-NC@ACC	70	0.2	~1114	1000	780	9
$Ti_3C_2T_x$(4 h)-GN	75	2	~800	1000	~576	10
S@Co-N/G	90	1	~926.5	500	681	11
CNT@TiO_{2-x}–S	~70	1	717	500	590	12
VO_2(P)-NCNT/S	/	1	~818	500	~450	13
$Sn_{0.063}MoO_3$-S	67.4	1	905.7	500	721.1	14

Supplementary Table 5. The binding energy of Li_2S_x on c-CoO and a-CoO surfaces (unit: eV).

Terms	c-CoO	a-CoO
**L_2S**	-3.813	-8.867
**Li_2S_2**	-3.852	-9.252
**Li_2S_4**	-3.391	-7.582
**Li_2S_8**	-3.652	-7.290

Supplementary references

1. Ravel, B. & Newville, M. ATHENA, ARTEMIS, HEPHAESTUS: data analysis for X-ray absorption spectroscopy using IFEFFIT. *J. Synchrotron Rad.* **12**, 537-541 (2005).
2. Wu, S. *et al.* Porous hollow carbon nanospheres embedded with well-dispersed cobalt monoxide nanocrystals as effective polysulfide reservoirs for high-rate and long-cycle lithium–sulfur batteries. *J. Mater. Chem. A* **5**, 17352-17359 (2017).
3. Chang, Z. *et al.* Co_3O_4 nanoneedle arrays as a multifunctional "super-reservoir" electrode for long cycle life Li–S batteries. *J. Mater. Chem. A* **5**, 250-257 (2017).
4. Wang, H. *et al.* Ultrathin Cobaltosic Oxide Nanosheets as an Effective Sulfur Encapsulation Matrix with Strong Affinity Toward Polysulfides. *ACS Appl. Mater. Interfaces* **9**, 4320-4325 (2017).
5. Li, Z., Zhang, J. & Lou, X.W. Hollow Carbon Nanofibers Filled with MnO_2 Nanosheets as Efficient Sulfur Hosts for Lithium-Sulfur Batteries. *Angew. Chem. Int. Ed.* **54**, 12886-12890 (2015).
6. Fan, Q. *et al.* Ternary Hybrid Material for High-Performance Lithium-Sulfur Battery. *J. Am. Chem. Soc.* **137**, 12946-12953 (2015).
7. Pang, Q., Kundu, D. & Nazar, L.F. A graphene-like metallic cathode host for long-life and high-loading lithium–sulfur batteries. *Mater. Horizons* **3**, 130-136 (2016).
8. Yuan, H. *et al.* Conductive and Catalytic Triple-Phase Interfaces Enabling Uniform Nucleation in High-Rate Lithium-Sulfur Batteries. *Adv. Energy Mater.* **9**, 1802768 (2019).
9. Lu, K. *et al.* Manipulating Polysulfide Conversion with Strongly Coupled Fe_3O_4 and Nitrogen Doped Carbon for Stable and High Capacity Lithium-Sulfur Batteries. *Adv. Funct. Mater.* **29**, 1807309 (2019).
10. Jiao, L. *et al.* Capture and Catalytic Conversion of Polysulfides by In Situ Built TiO_2-M Xene Heterostructures for Lithium-Sulfur Batteries. *Adv. Energy Mater.* **9**, 1900219 (2019).
11. Du, Z. *et al.* Cobalt in Nitrogen-Doped Graphene as Single-Atom Catalyst for High-Sulfur Content Lithium-Sulfur Batteries. *J. Am. Chem. Soc.* **141**, 3977-3985 (2019).
12. Wang, Y. *et al.* Enhancing Catalytic Activity of Titanium Oxide in Lithium–Sulfur Batteries by Band Engineering. *Adv. Energy Mater.* 1900953 (2019).
13. Wang, S. *et al.* Designing a highly efficient polysulfide conversion catalyst with paramontroseite for high-performance and long-life lithium-sulfur batteries. *Nano Energy* **57**, 230-240 (2019).
14. Yang, W. *et al.* Tin Intercalated Ultrathin MoO_3 Nanoribbons for Advanced Lithium-Sulfur Batteries. *Adv. Energy Mater.* **9**, 1803137 (2019).
